# Population genetics analysis of *Phlebotomus papatasi* sand flies from Egypt and Jordan based on mitochondrial cytochrome *b* haplotypes

**DOI:** 10.1186/s13071-018-2785-9

**Published:** 2018-03-27

**Authors:** Catherine M. Flanley, Marcelo Ramalho-Ortigao, Iliano V. Coutinho-Abreu, Rami Mukbel, Hanafi A. Hanafi, Shabaan S. El-Hossary, Emad El-Din Y. Fawaz, David F. Hoel, Alexander W. Bray, Gwen Stayback, Douglas A. Shoue, Shaden Kamhawi, Mehmet Karakuş, Kaouther Jaouadi, Mohammad Reza Yaghoobie-Ershadi, Andreas Krüger, Ahmad Amro, Mohamed Amin Kenawy, Mostafa Ramadhan Dokhan, Alon Warburg, Omar Hamarsheh, Mary Ann McDowell

**Affiliations:** 1 Eck Institute for Global Health, Department of Biological Sciences, University of Notre Dame, Notre Dame, IN 46556 USA; 2 F. Edward Hebert School of Medicine, Department of Preventive Medicine and Biostatistics, Uniformed Services University of the Health Sciences (USUHS), Bethesda, MD 20814 USA; 3 Laboratory of Malaria and Vector Research, NIAID-NIH, 12735 Twinbrook Parkway, Rockville, MD 20852 USA; 4 Faculty of Veterinary Medicine, Jordan University of Science and Technology, Irbid, 22110 Jordan; 5 Vector Biology Research Program, U.S. Naval Medical Research Unit No. 3 (NAMRU-3), Cairo, Egypt; 6 Department of Parasitology, Faculty of Medicine, Ege University, İzmir, Turkey; 7 Department of Medical Epidemiology, Laboratory of Transmission, Control and Immunobiology of Infections (LR11IPT02) Institut Pasteur de Tunis, 13 Place Pasteur BP-74, 1002 Tunis-Belvedere, Tunisia; 8 Department of Medical Entomology & Vector Control, School of Public Health, Tehran University of Medical Sciences, Tehran, 1417613151 Iran; 9 Department of Tropical Medicine, Military Hospital Hamburg, Bernhard-Nocht-Straße 74, 20359 Hamburg, Germany; 10 Faculty of Pharmacy, Al-Quds University, Jerusalem, Palestine; 11 Department of Entomology, Faculty of Science, Ain Shams University, Abbassia, Cairo, 11566 Egypt; 12 Department of Zoology, Faculty of Science, University of Sabratha, Sabratha, Libya; 13 Department of Microbiology and Molecular Genetics, the Kuvin Center for the Study of Infectious and Tropical Diseases, Faculty of Medicine, Hadassah Medical School, Hebrew University, Jerusalem, Israel; 14 Department of Biological Sciences, Faculty of Science and Technology, Al-Quds University, Jerusalem, Palestine

**Keywords:** *Phlebotomus papatasi*, Cytochrome *b*, Sand flies, mtDNA, Egypt, Jordan, Haplotypes, Genetic differentiation, Population genetics

## Abstract

**Background:**

*Phlebotomus papatasi* sand flies are major vectors of *Leishmania major* and phlebovirus infection in North Africa and across the Middle East to the Indian subcontinent. Population genetics is a valuable tool in understanding the level of genetic variability present in vector populations, vector competence, and the development of novel control strategies. This study investigated the genetic differentiation between *P. papatasi* populations in Egypt and Jordan that inhabit distinct ecotopes and compared this structure to *P. papatasi* populations from a broader geographical range**.**

**Methods:**

A 461 base pair (bp) fragment from the mtDNA cytochrome *b* (*cyt b*) gene was PCR amplified and sequenced from 116 individual female sand flies from Aswan and North Sinai, Egypt, as well as Swaimeh and Malka, Jordan. Haplotypes were identified and used to generate a median-joining network, *F*_*ST*_ values and isolation-by-distance were also evaluated. Additional sand fly individuals from Afghanistan, Iran, Israel, Jordan, Libya, Tunisia and Turkey were included as well as previously published haplotypes to provide a geographically broad genetic variation analysis.

**Results:**

Thirteen haplotypes displaying nine variant sites were identified from *P. papatasi* collected in Egypt and Jordan. No private haplotypes were identified from samples in North Sinai, Egypt, two were observed in Aswan, Egypt, four from Swaimeh, Jordan and two in Malka, Jordan. The Jordan populations clustered separately from the Egypt populations and produced more private haplotypes than those from Egypt. Pairwise *F*_*ST*_ values fall in the range 0.024–0.648.

**Conclusion:**

The clustering patterns and pairwise *F*_*ST*_ values indicate a strong differentiation between Egyptian and Jordanian populations, although this population structure is not due to isolation-by-distance. Other factors, such as environmental influences and the genetic variability in the circulating *Le. major* parasites, could possibly contribute to this heterogeneity. The present study aligns with previous reports in that pockets of genetic differentiation exists between populations of this widely dispersed species but, overall, the species remains relatively homogeneous.

**Electronic supplementary material:**

The online version of this article (10.1186/s13071-018-2785-9) contains supplementary material, which is available to authorized users.

## Background

Approximately 0.7–1.2 million cases of cutaneous leishmaniasis (CL) are reported each year [1]. CL results in damage to the dermis and subcutaneous tissues during infection and complications may arise due to opportunistic bacterial and fungal infections or HIV co-infection [2]. Although the resulting lesions typically self-heal, they leave behind visible life-long scars that trigger social stigmatization of young people in endemic areas, especially females [3–5]. *Phlebotomus papatasi* is the primary vector of *Leishmania major*, one of the causative agents of cutaneous leishmaniasis in the Old World, the Middle East, and North Saharan Desert. *Phlebotomus papatasi* also transmits viruses that cause febrile illness in humans, including sand fly fever Naples virus and sand fly fever Sicilian virus [6].

*Leishmania major* infects approximately 1–2.4% of *P. papatasi* sand flies in Egypt and up to 5.5% in Jordan depending on the season [7, 8]. Different rodent species have been implicated as the reservoir in these countries. In Egypt, the primary reservoir species is *Gerbillus pyramidum* whereas in Jordan, the typical rodent reservoir is *Psammomys obesus* [7, 9]. Approximately 20% of individuals living in at-risk areas in Egypt are infected each year as compared to Jordan where 80% of individuals tested positive for leishmaniain skin tests in hyperendemic regions [7, 8].

*Phlebotomus papatasi* boasts a wide geographical distribution ranging from southern Europe around the Mediterranean Sea, northern Africa, the Middle East, and into India. These sand flies are able to inhabit a variety of ecological niches from tropical climates to arid desert [10]. Like other sand fly species, they colonize human housing and animal dwellings including shelters and rodent burrows [11], which provide a safe haven for many sand fly species and are associated with a high risk of transmission due to easy access of reservoir species such as the sand rat, *P*. *obesus* [12]. Female sand flies opportunistically blood feed from a variety of vertebrate hosts such as humans, dogs, rabbits, chickens and even lizards. The sand flies’ potential to expand into novel geographical areas is enhanced by climate change [13, 14]. Considering the large geographical expanse this sand fly covers and their ability to feed off a plethora of food sources, both plant and animal, the need for vector control is of vital importance to curb *Leishmania* transmission in the Old World. Currently, there is no efficacious vaccine or cure and current treatments can be toxic, time- and cost-prohibitive to those afflicted by poverty and CL, and many people lack access to treatment for cultural reasons or distance from treatment facilities.

Research has shown that exposure to sand fly saliva exacerbates *Le. major* infections in mice but pre-exposure to saliva from uninfected sand fly bites protects mice and attenuates the infection outcome through a delayed-type hypersensitivity immune reaction [15, 16]. These studies highlight the potential of the salivary proteins, such as *P. papatasi* salivary protein 15 (PpSP15), to be used as a vaccine [17–21]. Given their geographical range and limited dispersal ability, it is expected that different *P. papatasi* populations would demonstrate greater genetic diversity complicating the development of a saliva targeted vaccine [22].

Population genetic studies on sand fly vectors provide knowledge concerning speciation, cryptic species, vector dispersal capabilities, population structure, vector competence, and adaptability to changing environmental conditions such as climate, topography and vegetation [23–26]. Although these genetic differences in the genome can be difficult to detect, mitochondrial DNA (mtDNA) genes are commonly employed in population genetics studies due to their inherent sensitivity [23, 25, 27]. These markers have been widely used for population genetics analyses of the New World leishmaniasis sand fly, *Lutzomyia longipalpis* [28, 29]. MtDNA, for example the cytochrome *b* (*cyt b*) gene, is maternally inherited, and its slow rate of silent mutations allow enough differentiation to be detected between closely related populations that are within close geographical vicinity or conversely are separated by large geographical distances [27, 30–32]. The underlying population structure and genetic variability within and among geographically distant populations may influence vectorial capacity thus having epidemiological implications warranting assessment of control strategies to prevent transmission of CL [12, 24].

A variety of molecular markers, in addition to *cyt b*, have been employed to determine genetic variability among *P. papatasi* populations. Genomic DNA markers and DNA microsatellites have been previously utilized and revealed evidence of population subdivision among this species [33, 34]. One study, using mtDNA *cyt b* analysis, observed genetic differentiation among widely separated *P. papatasi* populations [31]. Analysis of mitochondrial DNA is widely used for studying differences between closely related species and closely related populations within close geographical vicinity [31, 35]. In this study, we aimed to understand the population structure and genetic variability of *P. papatasi* in Egypt and Jordan that inhabit distinct ecotopes through *cyt b* sequence analysis and compared this structure to *P. papatasi* populations from a broader geographical range.

## Methods

### Sand flies

**Fig. 1:**
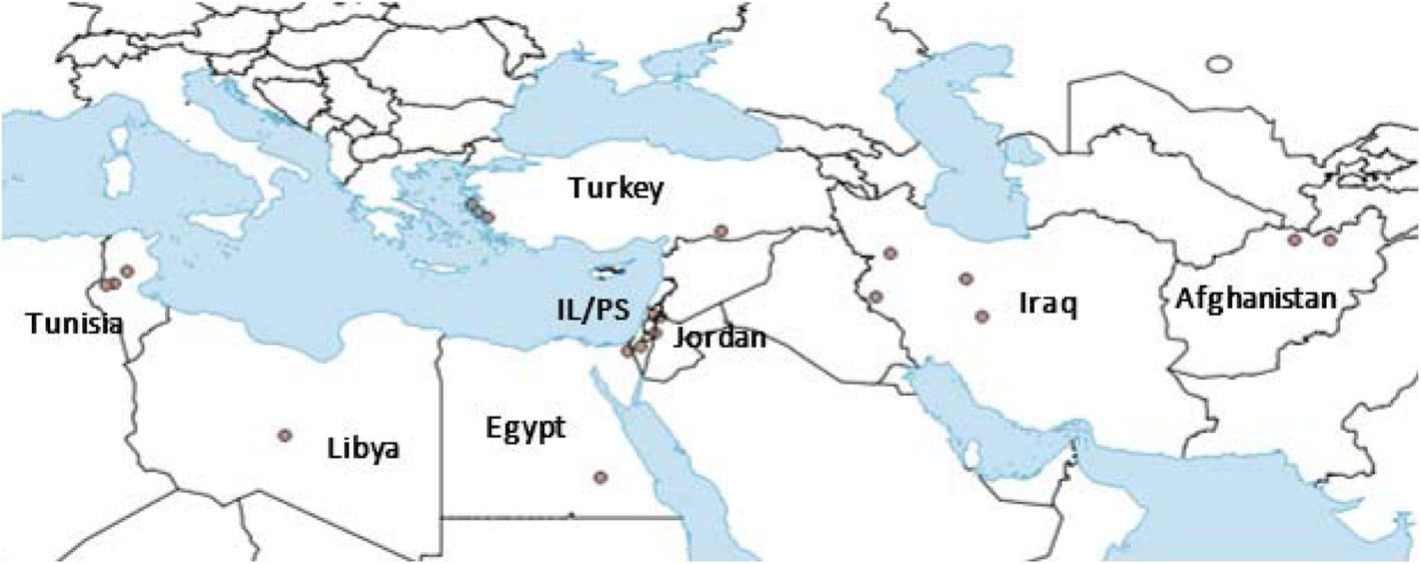
*Phlebotomus papatasi* population geographical locations. Libya study site is unknown; dot is used solely to indicate the country. *Abbreviation*: IL/PS, Israel/Palestine

*Phlebotomus papatasi* were collected (*n* = 133) from 21 field site and colony populations in 8 countries in 2006, 2007 and 2015 (Fig. 1, Table 1). Laboratory colonies were maintained at the University of Notre Dame (Israeli strain) and sent from the National Institutes of Health (Jordan strain). Field samples were collected from Afghanistan, Egypt, Iran, Jordan, Libya, Tunisia and Turkey. CDC light traps were used for *P. papatasi* collection. Sand flies were transported live to the laboratory and immediately processed or preserved specimens were sent to the University of Notre Dame. *Phlebotomus papatasi* males (the whole fly) and females (the whole fly except the head and last abdominal segments) were used for DNA extraction. Following the protocol outlined by Lane, *P. papatasi* were identified by microscopic examination of female spermateca [36].

**Table 1:** Study site geographical locations

Country	Population name	Code	*n*	Latitude	Longitude
Egypt and Jordan analysis					
Egypt	Aswan	AW	32	24.1667°N	32.8667°E
	North Sinai	NS	29	30.8333°N	34.1667°E
Jordan	Swaimeh	JS	26	31.8000°N	35.5833°E
	Malka	JM	29	32.6750°N	35.7492°E
Global analysis					
Afghanistan	Mazar-e-Sharif	AF	1	36.6926°N	67.1180°E
	Kunduz		1	36.7286°N	68.8681°E
Turkey	Kuşadası	TR	1	37.8579°N	27.2610°E
	Şanlıurfa		1	37.1674°N	38.7955°E
	Seferihisar		1	38.1951°N	26.8344°E
	Karaburun		1	38.6383°N	26.5127°E
Iran	Ilam	IN	1	33.6350°N	46.4153°E
	Qum		1	34.6399°N	50.8759°E
	Isfahan		1	32.6546°N	51.6680°E
	Kurdistan		1	35.9554°N	47.1362°E
Libya		LB	1		
Israel	Jordan Valley	IL	1	32.6933°N	35.4739°E
	Notre Dame		1		
Jordan	NIH	JO	1		
Tunisia	Ksar, Gafsa	TS	1	34.4274°N	8.8198°E
	Ouled Mhemed, Sidi Bozeid		1	35.0354°N	9.4839°E
	Al-Mitlawi, Gafsa		1	34.3194°N	8.4075°E

### DNA extraction

DNA was extracted using the Invitrogen PureLink® Genomic DNA Mini Kit (Invitrogen Corp., Carlsbad, CA) according to the manufacturer’s specifications. Individual male flies and the bodies of female flies were processed and DNA samples were stored at -20 °C.

### PCR amplification and sequencing

The 3'-end of the *cyt b* gene was amplified in 25 μl reaction volume: 10× Ex Taq Buffer (Takara Bio USA, Inc., Mountain View, CA), 2.5 mM dNTP mixture (Takara Bio USA, Inc., Mountain View, CA), 5 U/μl Ex Taq HS (Takara Bio USA, Inc., Mountain View, CA) and 10 μM each primer (CB3: 5'-CA(T/C) ATT CAA CC(A/T) GAA TGA TA-3' and N1N-PDR: 5'- GGT A(C/T)(A/T) TTG CCT CGA (T/A)TT CG(T/A) TAT GA-3') [30].

Samples were amplified in an Eppendorf Mastercycler (Eppendorf Inc., Enfield, CT) using the following protocol: denaturation at 95 °C for 5 min, followed by 10 cycles at 94 °C for 30 s, 40 °C for 30 s, 72 °C for 1.5 min, followed by 30 cycles at 94 °C for 30 s, 45 °C for 30 s, 72 °C for 1.5 min and a final elongation at 72 °C for 10 min. Samples were quantified and purity checked using Nanodrop™ Spectrophotometer (NanoDrop Technologies, Wilmington, DE).

Sequences were generated in the University of Notre Dame’s Genomics and Bioinformatics Core Facility using the DNA Sanger Sequencing Applied Biosystems 96-capillary 3730xl DNA Analyzer. Sequencing was carried out in both directions and the same primers were used. Sequences were edited using Geneious Pro 5.6.7 [37]. The *cyt b* sequences from 116 individual *P. papatasi* from Egypt and Jordan were analyzed. Twenty-one previously published, haplotypes available on GenBank [31] (accession numbers DQ381815-DQ381835), were included in this analysis as well as haplotypes derived from an additional 17 individual *P. papatasi* from field caught and colony populations originating in Afghanistan, Iran, Israel, Jordan, Libya, Tunisia and Turkey.

Multalin was used to align all *cyt b* sequences [38]. Gaps were treated as missing data, and haplotypes were generated from FASTA sequences using DnaSP software [39]. For median-joining analysis, Network 5 software [40] was used. Pairwise *F*_*ST*_ values and *F*_*ST*_/(1-*F*_*ST*_) values were calculated using Arlequin [41]. The corrected values were graphed *versus* geographical distance to elucidate possible isolation of populations by distance. Geographical distances were calculated using GPS coordinates.

## Results

### Egypt and Jordan analysis

A 461bp fragment from *P. papatasi cyt b* gene from 116 individual sand flies was PCR amplified and sequenced. The primers that hit the 3' end of *cyt b* were found to be an informative region and contain sequence variations that could be used for *P. papatasi* populations. This method has been in use since 1997 [42]. Sand flies were collected in four locations, two each in Egypt and Jordan. Number of flies collected per location were as follows: 32 in Aswan and 29 in North Sinai, Egypt; 26 in Swaimeh and 29 in Malka, Jordan. Alignment of the 116 individual *cyt b* fragment sequences indicated no insertions or deletions. The predicted amino acid sequences for all 116 fragments were obtained using the *Drosophila* mitochondrial code. A total of 13 haplotypes were identified (Table 2) with nine variant sites. All variant sites resulted from transition changes and no transversions have been identified. Four of the transitions were A→G and five were C→T. None of the variant sites were parsimony informative as only one type of nucleotide transition is present at each variant position. The haplotypes were comprised of a higher AT content (75.9%). The mean nucleotide diversity between all four study sites was 0.0021. Aswan and North Sinai demonstrated nucleotide diversity values of approximately 0.0010 whereas Swaimeh and Malka were approximately 0.0036.

**Table 2:** *Phlebotomus papatasi* private *cytochrome b* haplotypes

461 bp cyt *b* haplotype	Code	Frequency	Variant character position								
			0	0	1	1	2	2	2	2	4
			2	5	3	4	0	0	6	7	4
			7	7	2	4	1	4	7	6	1
PPH01	AW, NS, JS, JM	7	T	A	T	A	C	A	T	T	A
PPH04	AW, NS, JS, JM	61	.	.	.	G	.	.	.	.	.
PPH13	JS, JM	9	.	.	.	.	.	.	.	.	G
PPH22	AW	2	.	.	.	.	.	.	C	.	.
PPH23	AW	2	.	.	.	G	T	.	.	.	.
PPH24	JS	2	.	.	.	.	.	.	.	C	G
PPH25	JS	1	.	.	.	G	.	G	.	.	G
PPH26	JS, JM	7	C	.	.	.	.	.	.	.	.
PPH27	JS	2	.	.	C	.	.	.	.	.	.
PPH28	JS	1	C	.	.	.	.	.	.	.	G
PPH29	JM	1	.	G	.	.	.	.	.	.	.
PPH30	JM	1	C	G	.	.	.	.	.	.	.
PPH31	JS, JM	20	.	.	.	.	.	G	.	.	G

*Phlebotomus papatasi* private *cyt b* haplotypes from Aswan, Egypt (AW), North Sinai, Egypt (NS), Swaimeh, Jordan (JS) and Malka, Jordan (JM). 461-bp *cyt b* mtDNA gene. Alignment of variant positions. Dots indicate consensus with the first haplotype sequence, PPH01. PPHXX numbering is out of order to reflect the numbering of identical haplotypes in Fig. 4 and previously published haplotypes [31]

Haplotype PPH04 is the most frequent and found in all four populations investigated; Aswan, Egypt, North Sinai, Egypt, Swaimeh, Jordan and Malka, Jordan (Table 3). Haplotype PPH31 was the next most frequent haplotype and found only in *P. papatasi* populations from Jordan. Haplotype PPH01 was also found in all four populations but with lower frequency than PPH04. No private haplotypes were identified for the NS (Egypt) population, whereas two private haplotypes (PPH22 and PPH23) were found for AW (Egypt) population. The Jordanian collected *P. papatasi* exhibited six private haplotypes; four from Swaimeh (PPH24, PPH25, PPH27 and PPH28) and two from Malka (PPH29 and PPH30). Three haplotypes (PPH13, PPH26 and PPH31) were shared by both Jordan populations.

**Table 3:** *Cytochrome b* haplotype frequencies found in the Egypt and Jordan *P. papatasi* populations

Country	Population name	Code	Haplotypes PPHXX												
			01	04	13	22	23	24	25	26	27	28	29	30	31
Egypt	Aswan	AW	2	26		2	2								
	North Sinai	NS	1	28											
Jordan	Swaimeh	JS	2	1	2			2	1	3	2	1			12
	Malka	JM	2	6	7					4			1	1	8
Total			7	61	9	2	2	2	1	7	2	1	1	1	20

**Fig. 2:**
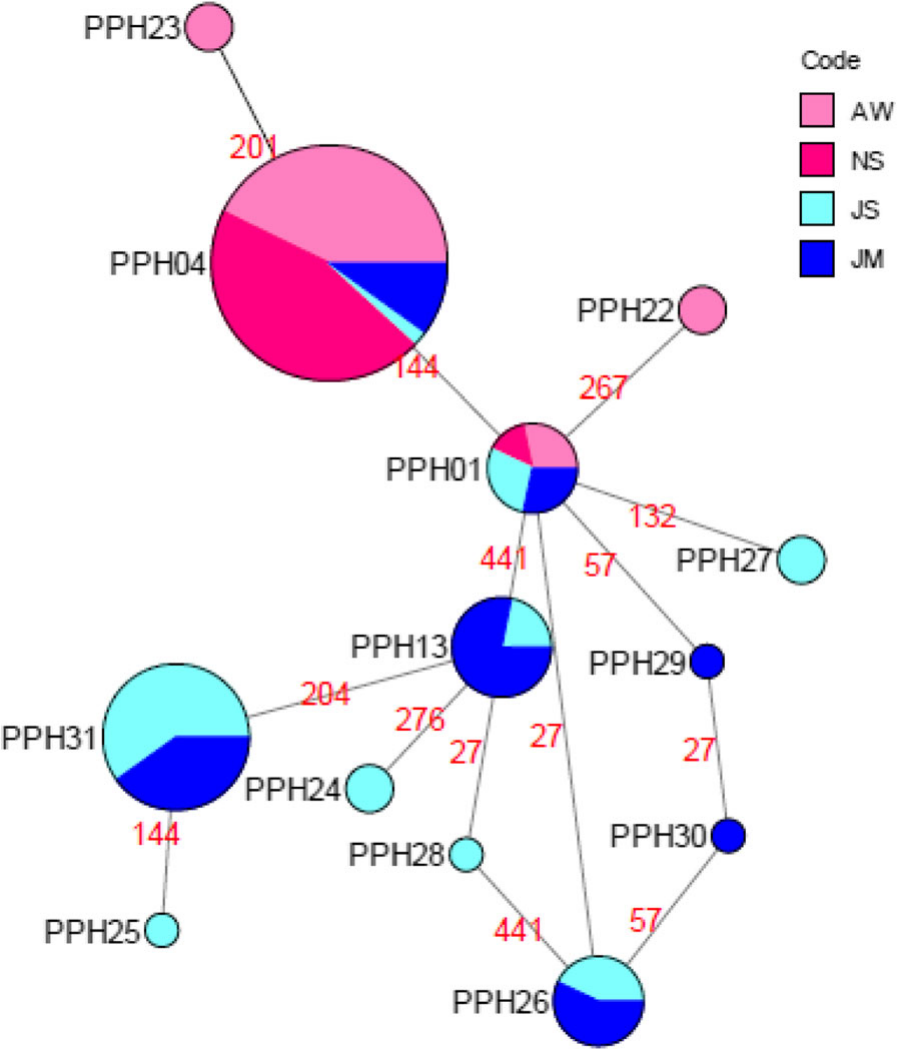
Median-joining network for 116 *Phlebotomus papatasi cyt b* sequences. Circle size and circle color indicates frequency and geographical location of haplotypes, respectively. Haplotype numbers are written next to the corresponding circle PPHXX. Red numbers between haplotypes indicate mutation sites. Older haplotypes are depicted as internal nodes while more recently diverged haplotypes depict the external nodes. PPHXX numbering is out of order to reflect the numbering of identical haplotypes in Fig. 4

A median-joining network of the 13 haplotypes depicts the relatedness between the haplotypes (Fig. 2). PPH01 is found at the center of the network, indicating it is the most ancestral haplotype and occurs in individuals from populations found at each study site. The haplotypes that comprise the external nodes of the network are more recently diverged than the internal nodes haplotypes. Only three nucleotide differences were found between the ancestral haplotype (PPH01) and all other haplotypes, while no more than five substitutions were identified between PPH04, which is the most frequent haplotype, and all other haplotypes.

**Fig. 3:**
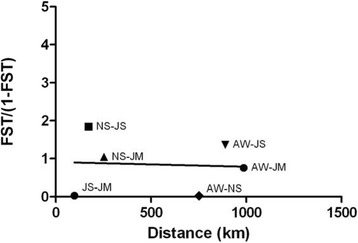
Isolation by distance estimate between four *P. papatasi* populations. *F*_*ST*_/(1-*F*_*ST*_) values compared to pairwise geographical distance

The number of private haplotypes from the two Jordan populations and their clustering in the median-joining network indicate significant genetic differentiation between populations of *P. papatasi* from Egypt and Jordan. Pairwise *F*_*ST*_ values reveal the highest (0.64815) between North Sinai, Egypt and Swaimeh, Jordan (Table 4). The lowest *F*_*ST*_ value (0.02402) occurred between Aswan and North Sinai, both in Egypt. There is also very little genetic differentiation between the two Jordan populations with an *F*_*ST*_ value of 0.03049. Comparisons between Egypt and Jordan populations indicate very great genetic differentiation, as defined by Wright [43]. No correlation was observed between *F*_*ST*_/(1-*F*_*ST*_) values and geographical distance (Fig. 3).

**Table 4:** *Cyt b* genetic differentiation estimates

Population	AW	NS	JS	JM
AW	0	ns	*	*
NS	0.02402	0	*	*
JS	0.57701	0.64815	0	ns
JM	0.43119	0.51076	0.03049	0

Values below the diagonal are pairwise *F*_*ST*_ values. Values above the diagonal indicate significance (*P < 0.01; ns, not significant)

### Global analysis

We compared previously described haplotypes [31] with the haplotypes identified in this study, as well as haplotypes generated from additional field and colony populations from Afghanistan, Iran, Israel, Jordan, Libya, Tunisia and Turkey. All of these sites taken together provide analysis that incorporates the broad geographical range of this important vector.

Thirty haplotypes were produced with 63 total substitutions at 34 variant sites (see Additional file 1: Table S1). Transitions accounted for 85.7% of the substitutions and 14.3% were transversions. There were equal numbers of A→G and C→T transitions. There were roughly equal numbers of transversions as well. There were 5 A→T transversions and 4 A→C. None of the substitution sites were parsimony informative.

The most frequent haplotype was PPH04 comprised of individuals from Afghanistan, Cyprus, Iran, Jordan, Tunisia, with the majority from Egypt (see Additional file 2: Table S2). PPH08 is the next most frequent haplotype that includes populations from Cyprus, Egypt, Israel, Palestine, Syria and Turkey. Six haplotypes that occur with the greatest frequencies (PPH01, PPH03, PPH04, PPH08, PPH13 and PPH31) are found in all populations included in this study except for Italy. Thirteen private haplotypes (PPH02, PPH05, PPH06, PPH10, PPH14, PPH15, PPH16, PPH19, PPH20, PPH25, PPH28, PPH29 and PPH30) were generated from Jordan, Turkey, Syria, Italy, Palestine and Egypt.

**Fig. 4:**
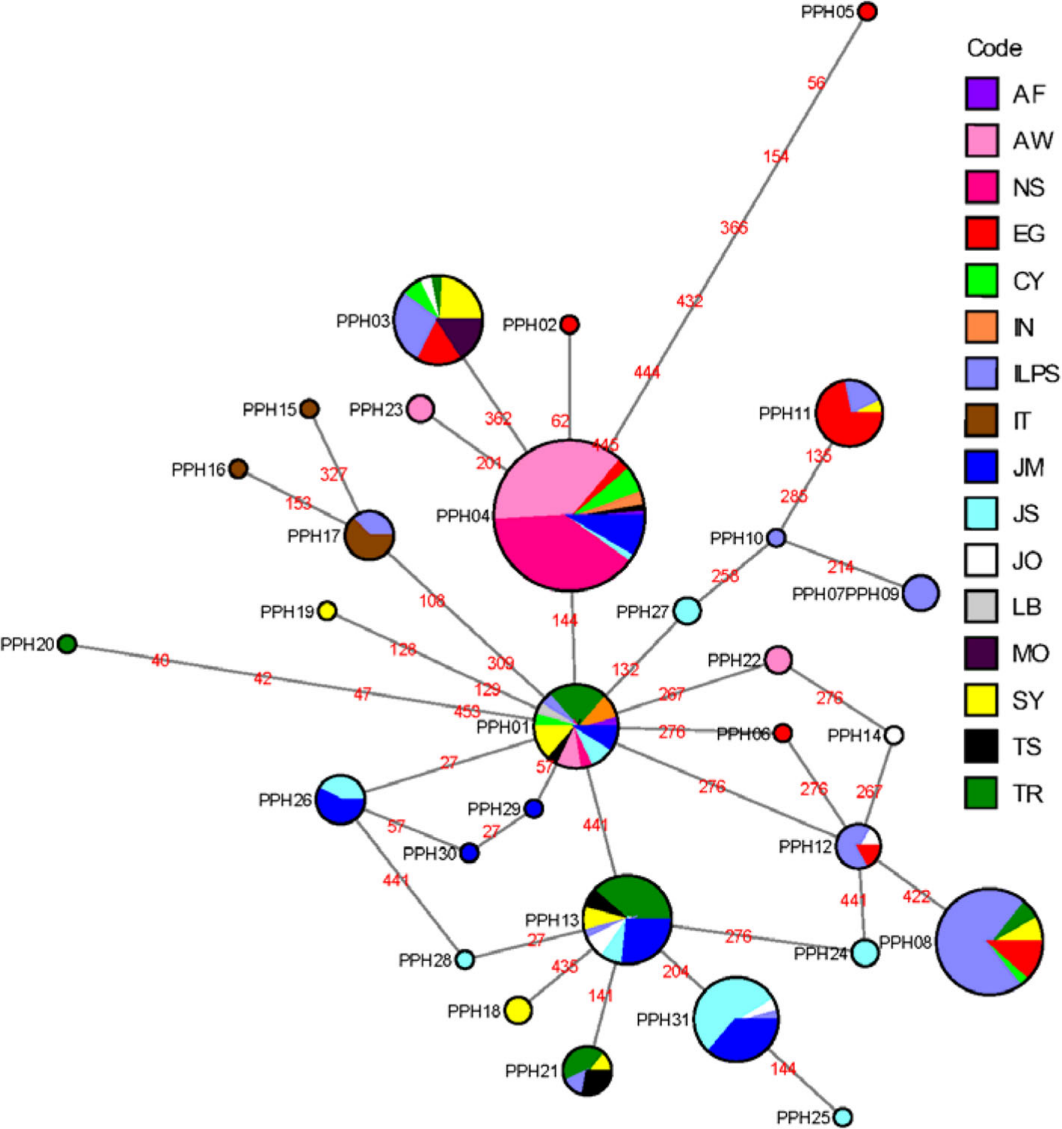
Global analysis median-joining network for 17 *Phlebotomus papatasi* populations. Circle size and circle color indicates frequency and geographical location of haplotypes, respectively. Haplotype numbers are written next to the corresponding circle PPHXX. Red numbers between haplotypes indicate mutation sites. Older haplotypes are depicted as internal nodes while more recently diverged haplotypes depict the external nodes. *Abbreviations*: CY, Cyprus; IL/PS, Israel/Palestine; SY, Syria; EG, Egypt; IT, Italy; MO, Morocco

A median-joining network for the 30 haplotypes was constructed to highlight the relationships between the haplotypes from these widely distributed populations (Fig. 4). PPH01 anchors the network as the most central, ancestral node. This haplotype is found in the majority of populations sampled (Afghanistan, Cyprus, Aswan, Egypt, North Sinai, Egypt, Iran, Israel, Malka, Jordan, Swaimeh, Jordan, Libya, Syria, Tunisia and Turkey). The median-joining network analysis demonstrates the same clustering patterns as seen in this study’s Egypt and Jordan analysis and with previously published studies [31, 44]. The Egypt and Jordan populations cluster separately as do the haplotypes circulating in the Israel and Palestine populations. PPH04 is the most frequent haplotype in the network. This haplotype includes mainly populations from Egypt. PPH03 diverged from PPH04 with one substitution between the two haplotypes. PPH03 includes populations from Palestine, Jordan, Cyprus, Turkey, Syria, Egypt and Morocco. The haplotypes found in populations circulating in Israel and Palestine also clustered together, as did the Jordan haplotypes; Italy also clustered independently from other regions. It is also important to note that there are few substitutions between the ancestral haplotype, PPH01, and the most recently diverged haplotypes, including rare haplotypes, located at the external nodes. The highest number of substitutions (7) occurs between PPH01 and PPH05.

## Discussion

This study ascertained the genetic variability of four *P. papatasi* populations from Egypt and Jordan using population genetics analysis of the *cyt b* gene. The median-joining network analysis of Egypt and Jordan populations identified PPH01 as the most ancestral haplotype. This haplotype is present and shared between all four populations and is located almost in the center of the network from which other haplotypes diverged by one or more mutational steps. The majority of Egypt individuals clustered together in PPH04, which is considered the most frequent haplotype in the network. Two private haplotypes (PPH22, PPH23) came from Aswan, Egypt. It was expected that the North Sinai individuals would cluster with the Jordanian samples from Swaimeh and Malka due to their close geographical proximity; approximately 170 and 250 km away, respectively. However, the Egypt populations and the Jordan populations clustered separately. More private haplotypes, occupying external node positions in the median-joining network, arose from Swaimeh, Jordan and Malka, Jordan, indicating these haplotypes are more recently diverged [45]. Previous work by Hamarsheh et al. [34] revealed the presence of two populations of *P. papatasi* from sand flies sampled around the Mediterranean Sea. More specifically, sand flies sampled from Egypt and Jordan clustered into population B, which further arranged into two subpopulations with no indication of geographical isolation [34]. This study is in agreement with previous work using *cyt b* analysis in that more haplotypes were present in the Jordan, Israel, Palestine region when compared to Egypt [31].

It is interesting to note that the number of private haplotypes present in *P. papatasi* from Jordan (*n* = 6) is greater than those from Egypt (*n* = 2); this may indicate that the Jordanian samples are more heterogeneous than those from Egypt. On the other hand, in Egypt, the Aswan samples look more heterogeneous than the North Sinai samples. The variation of topography, as well as differences between the North Sinai biome, which is a desert area with poor agricultural activity and the Aswan biome, which is more diverse and relies on the High Dam to provide water for extensive agriculture, may provide an explanation for the heterogeneous Aswan population.

The clear variation in clustering between Egypt and Jordan haplotypes suggest genetic differentiation between these populations. Indeed, pairwise *F*_*ST*_ comparisons reveal little genetic differentiation between Aswan and North Sinai in Egypt and Swaimeh and Malka in Jordan but exhibits large differentiation between the Egypt and Jordan study sites. The data suggest significant genetic differentiation between the Egypt and Jordan populations; however, this variation is not attributed to isolation by distance. This result was surprising, but not completely unexpected. As suggested by Khalid et al. [22], genetic differentiation detected between *P. papatasi* collected in Sudan and Egypt was not due to isolation-by-distance even though the study sites were approximately 1600 km apart. Here, as well as the previous study [20, 46, 47], ecological determinants such as the topography, climate, soil conditions and plant cover can influence the distribution of sand flies, specifically, *P. papatasi*. Moreover, anthropogenic changes can also lead to an increase in the dispersal of sand flies [22]. As a prolific peridomestic species, *P. papatasi* opportunistically inhabit human dwellings increasing the likelihood of disease transmission [6, 48, 49]. It is also possible that the genetic diversity between the Egypt and Jordan populations is influenced by distinct populations of *Le. major* circulating in the same geographical areas [50].

Microsatellite analysis demonstrated a moderate differentiation (*F*_*ST*_ *=* 0.1565) between the *Le. major* population in Egypt, ME1 and the Jordan population, ME2, possibly correlating with different native rodent species specific to Egypt and Jordan, as well as, greater biotope diversity in the Middle East [50]. A growing body of evidence continues to reinforce the idea that pathogen and vector genetic variability help shape this close relationship, ultimately driving vector competence [24, 51]. Similarly, isoenzyme analysis revealed that *Le. major* exhibits a high degree of homogeneity with predominantly two zymodemes circulating in the same geographical range as *P. papatasi* [52]. MON25 circulates in north-west Africa with MON26 dominates sub-Saharan Africa, north-east Africa and the Middle East. Although MON26 is the predominant zymodeme in Egypt and Jordan, two minor variants, MON74 and MON103, are also present. MON74 is observed in Africa and MON103 in the Middle East [52]. The minor *Le. major* zymodeme variants may contribute to the genetic diversity seen between the Egypt and Jordan *P. papatasi* populations as well. In a study from Iran, three subpopulations of *Le. major* coincide with three subpopulations of circulating *P. papatasi*, which may be due to topography and human movement [53]. Like other vector-parasite pairings in nature that share a long evolutionary history, *P. papatasi* and *Le. major* may be in a genetic arms-race [54, 55]. Although the mechanism is currently unknown, future studies concerning parasite-vector genetic variation may illuminate if coevolution or other forces influence genetic variation in the two species. Understanding the nuanced evolutionary relationship between *P. papatasi* and *Le. major* will inform *Leishmania* transmission and future control strategies [56].

Additional factors, such as altitude and/or varying rodent species at the different locals [10, 25, 48, 57] could affect the genetic differentiation between the Egypt and Jordan populations and offer an explanation why Jordan has more private haplotypes than Egypt. Aswan and North Sinai, Egypt, are located at altitudes of 117 m and 141 m above sea level, respectively. In Jordan, Swaimeh is located 345 m below sea level whereas Malka is located at 670 m above sea level. Such a difference in altitude between the two Jordanian trapping sites could account for the unequal number of private haplotypes between Swaimeh and Malka (Table 3) [58]. Although altitude itself does not solely determine ecotope traits, it does influence the vegetation available for *P. papatasi* sugar-feeding as well as potential reservoir species that harbor *Le. major* [25, 50, 53]. Other ecotope differences, such as climate, soil constituents and topography, in addition to different agricultural practices and land cover between Egypt and Jordan could also contribute to the genetic differentiation detected but need further study [23, 59].

Our work not only has an impact in scoring genetic variability between *P. papatasi* populations in Egypt and Jordan, but also in understanding how these populations fit into a more global view of this species’ genetic differentiation over a wide geographical range. In accordance with the analysis discussed above, the 30 haplotypes produced similar clustering patterns as observed for Egypt and Jordan as well as align with previously published reports [12, 44]. The Egypt and Jordan populations clustered separately as did the haplotypes circulating in Israel and Palestine populations. The anchoring, and most likely, ancestral node, PPH01, was represented in the majority of populations sampled including, Afghanistan, Cyprus, Egypt, Israel, Iran, Jordan, Libya, Syria, Tunisia and Turkey. The most frequent haplotypes in the network were shared by at least three or more populations. For example, PPH13 was shared by 7 populations and PPH08 is shared by 5, indicating species homogeneity.

This global analysis aligns with other studies [30, 33, 34, 44] demonstrating a relatively homogeneous population despite pockets of genetic variation seen in populations. The common thread connecting this body of literature to the current study is the fact that no clear phylogeographical pattern was observed indicating that although genetic variability exists when *P. papatasi* populations are compared, the species as a whole remains homogenous. Mitochondrial DNA analysis of the *nad*4 gene and the second internal transcribed spacer (ITS2) of the ribosomal DNA from 27 populations throughout the Mediterranean Basin, North Africa, Middle East and India, revealed no phylogeographical structure exhibiting molecular homogeneity as well [33]. Restricted gene flow, however, was indicated among populations from Turkey, Yemen, Egypt, Iran and Syria [33]. Using multi-locus microsatellite typing (MLMT) paired with Bayesian statistical analysis, Hamarsheh et al. [34], verified highly significant genetic variability between populations from northern Africa, the Middle East, southern Europe, India and Nepal. Even though genetic variability was detected, the overall population structure of *P. papatasi* exhibited two main populations implying species homogeneity [34]. Similar results were obtained by Raja et al. [44] when they analyzed *cyt b* in Tunisian populations of *P. papatasi*. Shared haplotypes were found at different nodes, including external nodes, in their analysis as well as reflected in our analysis with five haplotypes at external nodes (PPH03, PPH08, PPH11, PPH21 and PPH26) suggesting no phylogeographical pattern [44]. Esseghir et al. [30] analyzed the *cyt b* gene of 27 individual *P. papatasi* from 12 countries that produced 17 variant positions and 16 haplotypes. The majority of their haplotypes differed by 1–4 mutations with the highest number of mutations being six. Overall, they determined low genetic variability between wide spread *P. papatasi* populations. Similar mutational differences were observed in this paper as the majority of haplotypes differed by 1–4 mutations from the ancestral haplotype, with the most mutational steps being 7. Although, Esseghir et al. [30] reported fewer haplotypes and mutations compared to the present study, their sampling method may have resulted in lower mitochondrial diversity as 18 of their 27 samples were from colony-reared sand flies whereas the majority of sand flies sampled in this study were field collected. Overall, although localized genetic variation exists between populations, when *P. papatasi* populations are sampled over a wide geographical distribution, the species seems to be relatively homogenous making sand fly control strategies possible.

Recent population genetics studies, like the research presented here coupled with future studies that delve into the trifecta of vector-parasite-environment interactions are vital to expose what drives genetic differentiation, how vectorial competency is impacted and changes in vector and parasite dispersal dynamics [24, 51, 60, 61]. With a common goal to decrease the incidence of cutaneous leishmaniasis worldwide, basic biological research informs how best to achieve this goal through targeted vector control, vaccine development, or a combination of strategies.

## Conclusions

The present study confirms the presence of genetic variation between *P. papatasi* populations from Aswan and North Sinai, Egypt, and Swaimeh and Malka, Jordan, using the mitochondrial *cyt b* gene. Although the Egypt populations exhibit very great genetic differentiation when compared with the populations from Jordan, this is not due to isolation-by-distance. Other factors, such as genetic variability in the circulating *Le. major* parasites, other environmental conditions, and/or a variety of reservoir and intermediate hosts present for each study site, may contribute to the detected genetic differentiation. The present study aligns with the growing body of literature in that localized or pockets of genetic differentiation exists between the populations of this widely dispersed species but overall, the species remains relatively homogeneous. The continued surveying of sand fly population genetics helps monitor the dispersal dynamics and vector competency as well as informs the development of control strategies against this important *Le. major* vector.

## Additional files

Additional file 1: **Table S1*.*** *Phlebotomus papatasi* unique *cytochrome b* mt gene haplotypes (461 bp) from geographically distant populations. Alignment of variant positions. Dots indicate consensus with the first haplotype sequence, PPH01. (DOCX 21 kb)

Additional file 2: **Table S2.** *Cytochrome b* haplotype frequencies found in geographically distant *Phlebotomus papatasi* populations. (DOCX 16 kb)
