# Supplementary material for: Population genetics analysis of Phlebotomus papatasi sand flies from Egypt and Jordan based on mitochondrial cytochrome b haplotypes
Source: Parasit Vectors. 2018 Mar 27;11:214. doi: 10.1186/s13071-018-2785-9 (PMC5872541; doi:10.1186/s13071-018-2785-9)
Supplement: Supplementary file 1 — Table S1. Phlebotomus papatasi unique cytochrome b mt gene haplotypes (461 bp) from geographically distant populations. Alignment of variant positions. Dots indicate consensus with the first haplotype sequence, PPH01. (DOCX 21 kb) [file 13071_2018_2785_MOESM1_ESM.docx]

|  |  | Variant character position | | | | | | | | | | | | | | | | | | | | | | | | | | | | | | | | | |
| --- | --- | --- | --- | --- | --- | --- | --- | --- | --- | --- | --- | --- | --- | --- | --- | --- | --- | --- | --- | --- | --- | --- | --- | --- | --- | --- | --- | --- | --- | --- | --- | --- | --- | --- | --- |
| 461-bp |  | 0 | 0 | 0 | 0 | 0 | 0 | 0 | 1 | 1 | 1 | 1 | 1 | 1 | 1 | 1 | 1 | 2 | 2 | 2 | 2 | 2 | 2 | 2 | 3 | 3 | 3 | 3 | 4 | 4 | 4 | 4 | 4 | 4 | 4 |
| *Cyt b* |  | 2 | 4 | 4 | 4 | 5 | 5 | 6 | 0 | 2 | 2 | 3 | 3 | 4 | 4 | 5 | 5 | 0 | 0 | 1 | 5 | 6 | 7 | 8 | 0 | 2 | 6 | 6 | 2 | 3 | 3 | 4 | 4 | 4 | 5 |
| Haplotype | Frequency | 7 | 0 | 2 | 7 | 6 | 7 | 2 | 8 | 8 | 9 | 2 | 5 | 1 | 4 | 3 | 4 | 1 | 4 | 4 | 8 | 7 | 6 | 5 | 9 | 7 | 2 | 6 | 2 | 2 | 5 | 1 | 4 | 5 | 3 |
| PPH01 | 22 | T | C | T | A | G | A | T | C | G | A | T | A | C | A | A | A | C | A | G | T | T | T | A | C | A | G | G | T | A | C | A | A | A | A |
| PPH02 | 1 | . | . | . | . | . | . | C | . | . | . | . | . | . | G | . | . | . | . | . | . | . | . | . | . | . | . | . | . | . | . | . | . | . | . |
| PPH03 | 25 | . | . | . | . | . | . | . | . | . | . | . | . | . | G | . | . | . | . | . | . | . | . | . | . | . | A | . | . | . | . | . | . | . | . |
| PPH04 | 71 | . | . | . | . | . | . | . | . | . | . | . | . | . | G | . | . | . | . | . | . | . | . | . | . | . | . | . | . | . | . | . | . | . | . |
| PPH05 | 1 | . | . | . | . | A | . | . | . | . | . | . | . | . | G | . | G | . | . | . | . | . | . | . | . | . | . | A | . | C | . | . | C | T | . |
| PPH06 | 1 | . | . | . | . | . | . | . | . | . | . | . | . | . | . | . | . | . | . | . | . | . | A | . | . | . | . | . | . | . | . | . | . | . | . |
| PPH07/09 | 4 | . | . | . | . | . | . | . | . | . | . | . | . | . | . | . | . | . | . | A | C | . | . | . | . | . | . | . | . | . | . | . | . | . | . |
| PPH08 | 35 | . | . | . | . | . | . | . | . | . | . | . | . | . | . | . | . | . | . | . | . | . | C | . | . | . | . | . | A | . | . | . | . | . | . |
| PPH10 | 1 | . | . | . | . | . | . | . | . | . | . | C | . | . | . | . | . | . | . | . | C | . | . | . | . | . | . | . | . | . | . | . | . | . | . |
| PPH11 | 14 | . | . | . | . | . | . | . | . | . | . | C | G | . | . | . | . | . | . | . | C | . | . | T | . | . | . | . | . | . | . | . | . | . | . |
| PPH12 | 6 | . | . | . | . | . | . | . | . | . | . | . | . | . | . | . | . | . | . | . | . | . | C | . | . | . | . | . | . | . | . | . | . | . | . |
| PPH13 | 26 | . | . | . | . | . | . | . | . | . | . | . | . | . | . | . | . | . | . | . | . | . | . | . | . | . | . | . | . | . | . | G | . | . | . |
| PPH14 | 1 | . | . | . | . | . | . | . | . | . | . | . | . | . | . | . | . | . | . | . | . | C | C | . | . | . | . | . | . | . | . | . | . | . | . |
| PPH15 | 1 | . | . | . | . | . | . | . | T | . | . | . | . | . | . | . | . | . | . | . | . | . | . | . | T | G | . | . | . | . | . | . | . | . | . |
| PPH16 | 1 | . | . | . | . | . | . | . | T | . | . | . | . | . | . | G | . | . | . | . | . | . | . | . | T | . | . | . | . | . | . | . | . | . | . |
| PPH17 | 8 | . | . | . | . | . | . | . | T | . | . | . | . | . | . | . | . | . | . | . | . | . | . | . | T | . | . | . | . | . | . | . | . | . | . |
| PPH18 | 2 | . | . | . | . | . | . | . | . | . | . | . | . | . | . | . | . | . | . | . | . | . | . | . | . | . | . | . | . | . | T | G | . | . | . |
| PPH19 | 1 | . | . | . | . | . | . | . | . | A | G | . | . | . | . | . | . | . | . | . | . | . | . | . | . | . | . | . | . | . | . | . | . | . | . |
| PPH20 | 1 | . | A | C | T | . | . | . | . | . | . | . | . | . | . | . | . | . | . | . | . | . | . | . | . | . | . | . | . | . | . | . | . | . | C |
| PPH21 | 7 | . | . | . | . | . | . | . | . | . | . | . | . | T | . | . | . | . | . | . | . | . | . | . | . | . | . | . | . | . | . | G | . | . | . |
| PPH22 | 2 | . | . | . | . | . | . | . | . | . | . | . | . | . | . | . | . | . | . | . | . | C | . | . | . | . | . | . | . | . | . | . | . | . | . |
| PPH23 | 2 | . | . | . | . | . | . | . | . | . | . | . | . | . | G | . | . | T | . | . | . | . | . | . | . | . | . | . | . | . | . | . | . | . | . |
| PPH24 | 2 | . | . | . | . | . | . | . | . | . | . | . | . | . | . | . | . | . | . | . | . | . | C | . | . | . | . | . | . | . | . | G | . | . | . |
| PPH25 | 1 | . | . | . | . | . | . | . | . | . | . | . | . | . | G | . | . | . | G | . | . | . | . | . | . | . | . | . | . | . | . | G | . | . | . |
| PPH26 | 7 | C | . | . | . | . | . | . | . | . | . | . | . | . | . | . | . | . | . | . | . | . | . | . | . | . | . | . | . | . | . | . | . | . | . |
| PPH27 | 2 | . | . | . | . | . | . | . | . | . | . | C | . | . | . | . | . | . | . | . | . | . | . | . | . | . | . | . | . | . | . | . | . | . | . |
| PPH28 | 1 | C | . | . | . | . | . | . | . | . | . | . | . | . | . | . | . | . | . | . | . | . | . | . | . | . | . | . | . | . | . | G | . | . | . |
| PPH29 | 1 | . | . | . | . | . | G | . | . | . | . | . | . | . | . | . | . | . | . | . | . | . | . | . | . | . | . | . | . | . | . | . | . | . | . |
| PPH30 | 1 | C | . | . | . | . | G | . | . | . | . | . | . | . | . | . | . | . | . | . | . | . | . | . | . | . | . | . | . | . | . | . | . | . | . |
| PPH31 | 22 | . | . | . | . | . | . | . | . | . | . | . | . | . | . | . | . | . | G | . | . | . | . | . | . | . | . | . | . | . | . | G | . | . | . |

**Additional File 1: Table S1***. Phlebotomus papatasi* unique *cytochrome b* haplotypes from geographically distant populations.

461-bp *cytochrome b* mt gene. Alignment of variant positions. Dots indicate consensus with the first haplotype sequence, PPH01.
