# Supplementary material for: Population genetics analysis of Phlebotomus papatasi sand flies from Egypt and Jordan based on mitochondrial cytochrome b haplotypes
Source: Parasit Vectors. 2018 Mar 27;11:214. doi: 10.1186/s13071-018-2785-9 (PMC5872541; doi:10.1186/s13071-018-2785-9)
Supplement: Supplementary file 2 — Table S2. Cytochrome b haplotype frequencies found in geographically distant Phlebotomus papatasi populations. (DOCX 16 kb) [file 13071_2018_2785_MOESM2_ESM.docx]

|  |  | Haplotypes PPHXX | | | | | | | | | | | | | | | | | | | | | | | | | | | | | |
| --- | --- | --- | --- | --- | --- | --- | --- | --- | --- | --- | --- | --- | --- | --- | --- | --- | --- | --- | --- | --- | --- | --- | --- | --- | --- | --- | --- | --- | --- | --- | --- |
| Country | Population Name | 01 | 02 | 03 | 04 | 05 | 06 | 07/09 | 08 | 10 | 11 | 12 | 13 | 14 | 15 | 16 | 17 | 18 | 19 | 20 | 21 | 22 | 23 | 24 | 25 | 26 | 27 | 28 | 29 | 30 | 31 |
| Afghanistan | Mazar-e-Sharif | 1 |  |  |  |  |  |  |  |  |  |  |  |  |  |  |  |  |  |  |  |  |  |  |  |  |  |  |  |  |  |
|  | Kunduz |  |  |  | 1 |  |  |  |  |  |  |  |  |  |  |  |  |  |  |  |  |  |  |  |  |  |  |  |  |  |  |
| Cyprus | * | 1 |  | 2 | 4 |  |  |  | 1 |  |  |  |  |  |  |  |  |  |  |  |  |  |  |  |  |  |  |  |  |  |  |
| Egypt | * |  | 1 | 4 | 2 | 1 | 1 |  | 4 |  | 10 | 1 |  |  |  |  |  |  |  |  |  |  |  |  |  |  |  |  |  |  |  |
|  | Aswan | 2 |  |  | 26 |  |  |  |  |  |  |  |  |  |  |  |  |  |  |  |  | 2 | 2 |  |  |  |  |  |  |  |  |
|  | North Sinai | 1 |  |  | 28 |  |  |  |  |  |  |  |  |  |  |  |  |  |  |  |  |  |  |  |  |  |  |  |  |  |  |
| Iran | Ilam | 1 |  |  |  |  |  |  |  |  |  |  |  |  |  |  |  |  |  |  |  |  |  |  |  |  |  |  |  |  |  |
|  | Qum |  |  |  | 1 |  |  |  |  |  |  |  |  |  |  |  |  |  |  |  |  |  |  |  |  |  |  |  |  |  |  |
|  | Isfahan |  |  |  | 1 |  |  |  |  |  |  |  |  |  |  |  |  |  |  |  |  |  |  |  |  |  |  |  |  |  |  |
|  | Kurdistan | 1 |  |  |  |  |  |  |  |  |  |  |  |  |  |  |  |  |  |  |  |  |  |  |  |  |  |  |  |  |  |
| Israel | * |  |  |  |  |  |  | 3 | 1 |  |  |  |  |  |  |  |  |  |  |  |  |  |  |  |  |  |  |  |  |  |  |
|  | Jordan Valley |  |  |  |  |  |  |  |  |  |  |  |  |  |  |  |  |  |  |  |  |  |  |  |  |  |  |  |  |  |  |
|  | Notre Dame colony | 1 |  |  |  |  |  |  |  |  |  |  |  |  |  |  |  |  |  |  |  |  |  |  |  |  |  |  |  |  | 1 |
| Italy | * |  |  |  |  |  |  |  |  |  |  |  |  |  | 1 | 1 | 5 |  |  |  |  |  |  |  |  |  |  |  |  |  |  |
| Jordan | * |  |  | 1 |  |  |  |  |  |  |  | 1 | 2 | 1 |  |  |  |  |  |  |  |  |  |  |  |  |  |  |  |  |  |
|  | Swaymeh | 2 |  |  | 1 |  |  |  |  |  |  |  | 2 |  |  |  |  |  |  |  |  |  |  | 2 | 1 | 3 | 2 | 1 |  |  | 12 |
|  | Malka | 2 |  |  | 6 |  |  |  |  |  |  |  | 7 |  |  |  |  |  |  |  |  |  |  |  |  | 4 |  |  | 1 | 1 | 8 |
|  | NIH colony |  |  |  |  |  |  |  |  |  |  |  |  |  |  |  |  |  |  |  |  |  |  |  |  |  |  |  |  |  | 1 |
| Libya | Libya | 1 |  |  |  |  |  |  |  |  |  |  |  |  |  |  |  |  |  |  |  |  |  |  |  |  |  |  |  |  |  |
| Morocco | * |  |  | 4 |  |  |  |  |  |  |  |  |  |  |  |  |  |  |  |  |  |  |  |  |  |  |  |  |  |  |  |
| Palestine | * |  |  | 7 |  |  |  | 1 | 24 | 1 | 3 | 4 | 1 |  |  |  | 3 |  |  |  | 1 |  |  |  |  |  |  |  |  |  |  |
| Syria | * | 3 |  | 6 |  |  |  |  | 3 |  | 1 |  | 2 |  |  |  |  | 2 | 1 |  | 1 |  |  |  |  |  |  |  |  |  |  |
| Tunisia | * | 1 |  |  |  |  |  |  |  |  |  |  |  |  |  |  |  |  |  |  | 2 |  |  |  |  |  |  |  |  |  |  |
|  | Ksar, Gafsa |  |  |  |  |  |  |  |  |  |  |  | 1 |  |  |  |  |  |  |  |  |  |  |  |  |  |  |  |  |  |  |
|  | Ouled Mhemed, Sidi Bozeid |  |  |  |  |  |  |  |  |  |  |  | 1 |  |  |  |  |  |  |  |  |  |  |  |  |  |  |  |  |  |  |
|  | Al-Mitlawi, Gafsa |  |  |  | 1 |  |  |  |  |  |  |  |  |  |  |  |  |  |  |  |  |  |  |  |  |  |  |  |  |  |  |
| Turkey | * | 2 |  |  |  |  |  |  | 2 |  |  |  | 9 |  |  |  |  |  |  | 1 | 3 |  |  |  |  |  |  |  |  |  |  |
|  | Kuşadası | 1 |  |  |  |  |  |  |  |  |  |  |  |  |  |  |  |  |  |  |  |  |  |  |  |  |  |  |  |  |  |
|  | Şanlıurfa |  |  |  |  |  |  |  |  |  |  |  | 1 |  |  |  |  |  |  |  |  |  |  |  |  |  |  |  |  |  |  |
|  | Seferihisar | 1 |  |  |  |  |  |  |  |  |  |  |  |  |  |  |  |  |  |  |  |  |  |  |  |  |  |  |  |  |  |
|  | Karaburun | 1 |  |  |  |  |  |  |  |  |  |  |  |  |  |  |  |  |  |  |  |  |  |  |  |  |  |  |  |  |  |
|  |  |  |  |  |  |  |  |  |  |  |  |  |  |  |  |  |  |  |  |  |  |  |  |  |  |  |  |  |  |  |  |
|  | Totals: | 22 | 1 | 25 | 71 | 1 | 1 | 4 | 35 | 1 | 14 | 6 | 26 | 1 | 1 | 1 | 8 | 2 | 1 | 1 | 7 | 2 | 2 | 2 | 1 | 7 | 2 | 1 | 1 | 1 | 22 |
| * Previously published in Hamarsheh *et al*. (2007) | | | | | | | | | | | | | | | | | | | | | | | | | | | | | | | |

**Additional File 2: Table S2**. *Cytochrome b* haplotype frequencies found in geographically distant *Phlebotomus papatasi* populations.
